# Hybrid UV-Ozone-Treated rGO-PEDOT:PSS as an Efficient Hole Transport Material in Inverted Planar Perovskite Solar Cells

**DOI:** 10.1186/s11671-017-2393-1

**Published:** 2017-12-13

**Authors:** Shuying Wang, Xiaona Huang, Haoxuan Sun, Chunyang Wu

**Affiliations:** 1 State Key Laboratory of Electronic Thin Films and Integrated Devices, University of Electronic Science and Technology of China, Chengdu, 610054 China; 2 Chengdu NO.7 High School, Gaoxin Campus, Chengdu, 610041 China; 3 Chengdu Technology University, Chengdu, 611730 China

**Keywords:** Inverted planar perovskite solar cells, Reduced graphene oxide (rGO), UV-ozone treatment, PEDOT:PSS

## Abstract

Inverted planar perovskite solar cells (PSCs), which are regarded as promising devices for new generation of photovoltaic systems, show many advantages, such as low-temperature film formation, low-cost fabrication, and smaller hysteresis compared with those of traditional n-i-p PSCs. As an important carrier transport layer in PSCs, the hole transport layer (HTL) considerably affects the device performance. Therefore, HTL modification becomes one of the most critical issues in improving the performance of PSCs. In this paper, we report an effective and environmentally friendly UV-ozone treatment method to enhance the hydrophilia of reduced graphene oxide (rGO) with its excellent electrical performance. The treated rGO was applied to doped poly(3,4-ethylenedioxythiophene) poly(styrene-sulfonate) (PEDOT:PSS) as HTL material of PSCs. Consequently, the performance of rGO/PEDOT:PSS-doped PSCs was improved significantly, with power conversion efficiency (PCE) of 10.7%, *J*sc of 16.75 mA/cm^2^, *V*oc of 0.87 V, and FF of 75%. The PCE of this doped PSCs was 27% higher than that of the PSCs with pristine PEDOT:PSS as HTL. This performance was attributed to the excellent surface morphology and optimized hole mobility of the solution-processable rGO-modified PEDOT:PSS.

## Background

As one of the world’s top 10 scientific and technological breakthroughs in 2013, hybrid organic–inorganic perovskite material is regarded as one of the most promising materials for developing high-efficient photovoltaic devices because of its excellent photoelectric property [1–3]. In the past 7 years, the power conversion efficiency (PCE) of perovskite solar cells (PSCs) has increased remarkably from 3.8 to 22.1%, which outperforms those of conventional polycrystalline silicon solar cells [4]. Unfortunately, the traditional manufacture of n-i-p-type PSCs involving high-temperature annealing procedure is unavailable to flexible substrates, thereby limiting its commercial prospects. The novel solar cell device, which was first fabricated by Guo et al. in 2013 and delivered a PCE 3.9% [5], consists of poly(3,4-ethylenedioxythiophene) poly(styrene-sulfonate) (PEDOT:PSS) as the hole transport layer (HTL) and [6,6]-phenyl C61-butyric acid methyl ester as electron transport layer (ETL). Specifically, the p-type hole transport material (HTM) is deposited prior to the perovskite light absorption film. Subsequently, the n-type ETL is deposited onto the perovskite film. This p-i-n architecture is an inverted structure, which shows many excellent properties, such as easy fabrication, cost-effectiveness, small hysteresis, and high fill factor, compared with those of traditional n-i-p devices [6–8]. To date, the inverted planar PCSs have attracted considerable interest [9, 10]. Researchers used a variety of methods, including structure optimization [11–13], development of improved HTL [13] and ETL [14, 15], crystalline and morphology control of perovskites [16, 17], and interfacial engineering [18–20], to improve the efficiency of inverted PSCs. Unfortunately, the efficiency of inverted planar solar cells is still lower than that of traditional structure [21].

Graphene is a kind of 2D carbon nanomaterial that is composed of sp^2^-hybridized carbon atoms in a hexagonal structure [22]. This material possesses excellent advantages in electrical conductivity, optical transparency, and environment stability [23, 24]. HTL modification is one of the most important issues for improving the performance of inverted planar PSCs. For example, Yeo et al. applied reduced graphene oxide (rGO) nanosheets as HTLs, and the rGO-basic solar cell depicted a superior device efficiency (10.8%) to PEDOT:PSS- and GO-based solar cells [25]. Jokar et al. discussed the charge-extraction behavior of GO and rGO as p-contact layers for PSCs, demonstrating that the rGO synthesized via GO reduction with reducing agents obtains high-performance inverted planar heterojunction PSCs [26]. Moreover, graphene materials can serve as remarkable dopants to modify charge transport layer due to their long hot-electron lifetimes and ultrafast hot-electron extraction properties [27]. However, commercial graphene materials, such as rGO, aggregate when dispersed in water because of the absence of hydrophilic functional groups. GO exhibits low conductivity due to the damaged conjugated structure. Thus, a solution-processable graphene possessing both excellent electrical properties, such as rGO, and good dispersion characteristics, such as GO, should be well manufactured for HTL modification.

In this paper, we present a simple and environmentally friendly UV-ozone treatment method to obtain water-dispersed graphene with high charge mobility. Furthermore, we doped PEDOT:PSS using the obtained graphene to form an improved HTM in inverted PSCs. The incorporation of treated graphene into PEDOT:PSS increased the short-circuit current density and PCE of the PSCs. A remarkably enhanced *V*oc of 0.87 V with a relatively high *J*_SC_ of 16.75 mA/cm^2^ was obtained. The corresponding PCE with an average value of 10.75% was achieved with high reproducibility. The typical PCE of PSCs with rGO/PEDOT:PSS was improved by 27% compared with that of PSCs with pristine PEDOT:PSS as HTL.

## Methods/Experimental

### Chemicals

PEDOT:PSS (CleviosTM PVP. Al 4083) and CH_3_NH_3_I (MAI) were purchased from Heraeus Materials Technology Shanghai Ltd. and Deysol Ltd., respectively. PbI_2_ (99%), anhydrous *N*,*N*-dimethylformamide (DMF, 99.8%), and anhydrous chlorobenzene (CB, 99.8%) were supplied by Sigma-Aldrich company. [6,6]-Phenyl-C_61_-butyric acid methyl ester (PC61BM, > 99%) and 2,9-dimethyl-4,7-diphenyl-1,10-phenanthroline (BCP, > 99%) were obtained from Xi’an Polymer Light Technology Corp. rGO was synthesized by Y.F. Chen’s Team [28].

### Solution Preparation

Approximately 5 mg of rGO was placed in a quartz Petri dish and subsequently treated with UV-ozone cleaning procedure (operating power, 270 W) continuously for 2 h. Afterward, the obtained rGO was collected and added into deionized water to form a solution with the concentration of 1 mg/mL under ultrasonic bath treatment.

To obtain improved HTM for inverted PSCs, rGO solutions with different volume ratios (0.1, 0.2, and 0.3) were added into PEDOT:PSS solution at room temperature. The resultant rGO/PEDOT:PSS solutions were magnetically stirred overnight and filtered by polytetrafluoroethylene (PTFE) filters (0.45 μm).

The perovskite precursor solution was prepared by the following processes. MAI and PbI_2_ powder were mixed in anhydrous DMF with a molar ratio of 1:1. Subsequently, the solution (40 wt%) was stirred overnight at 60 °C and filtered with 0.45-μm PTFE filters prior to device fabrication.

### Device Fabrication

The structure of the inverted planar heterojunction PSCs was indium tin oxide (ITO)/PEDOT:PSS/CH_3_NH_3_PbI_3_/PC_61_BM/BCP/Ag. The ITO substrate (1.5 × 1.5 cm^2^) was cleaned sequentially with acetone, isopropanol, and deionized water. The prepared UV-ozone-treaded rGO/PEDOT:PSS solution was spin coated to film at 4000 rpm for 40 s and thermally treated at 150 °C for 10 min in air. In this treatment, the perovskite active layer was deposited by one-step solution method through spin coating CH_3_NH_3_PbI_3_ precursor solution (40 wt% in DMF) at 4000 rpm for 40 s. To improve the crystallization of the active layer, 70 μL of CB was dropped quickly onto CH_3_NH_3_PbI_3_ wet film at approximately 6 s after the beginning of spinning, as reported in the literature [29]. The films were annealed at 110 °C for 30 min inside the glove box filled with nitrogen. Afterward, a solution of PC61BM in CB (20 mg/mL) was spin coated onto the perovskite film at 3000 rpm for 40 s. Subsequently, saturated BCP solution was spin coated in isopropyl alcohol at 2000 rpm for 30 s. Finally, a Ag layer (100 nm) was deposited by thermal evaporation.

### Characterization

The component analysis of rGO was conducted by X-ray photoelectron spectroscopy (XPS) with an ESCALAB 250 electron spectrometer. The crystallization structures of CH_3_NH_3_PbI_3_ layers were determined by X-ray diffraction (XRD Bede multifunctional high-resolution X-ray diffractometer, British). The film morphology was observed by atomic force microscopy (AFM, SPI3800, Japan). The current density–voltage (J–V) measurement was carried out by using Keithley model 2400 Source Meter under simulated AM 1.5 G solar illumination (100 mW/cm^2^) generated by solar simulator (ABET Technologies, SUN 3000).

## Results and Discussion

**Fig. 1:**
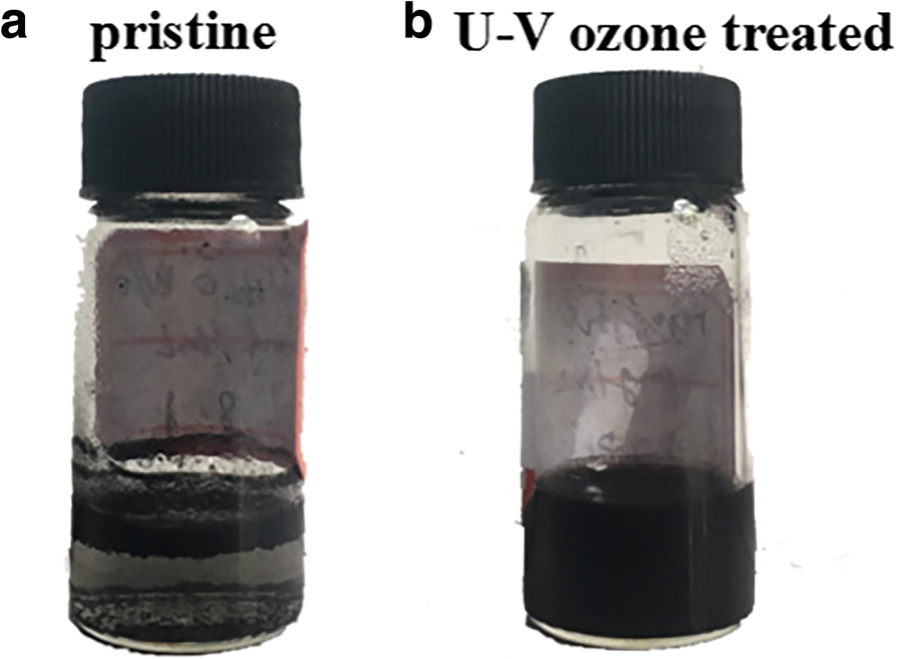
Photographs of **a** untreated and **b** UV-ozone-treated rGO solution (1 mg/mL in H_2_O)

The untreated and UV-ozone-treated rGOs dissolved in deionized water with the concentration of 1 mg/mL are shown in Fig. 1. The untreated rGO can be hardly dispersed in deionized water, and the treated one can be homogeneously dispersed in water, which is attributed to some –OH and –COOH groups in rGO. The UV-ozone-treated rGO solution still shows a deep black color compared with the brown 1 mg/mL commercial GO solution [22], thereby indicating the incomplete oxidation process of UV-ozone treatment.

**Fig. 2:**
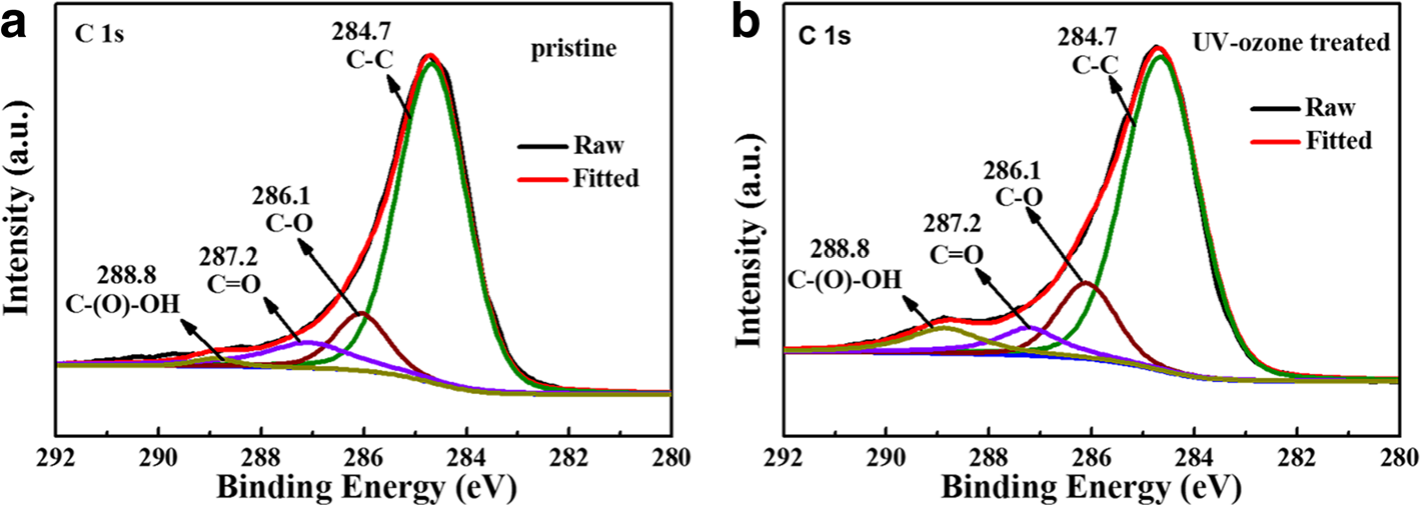
XPS spectra of **a** untreated and **b** UV-ozone-treated rGO

XPS measurement was performed to verify whether parts of the oxygen-containing groups of rGO underwent hydrophilic treatment. As shown in Fig. 2a, the C1s spectra of untreated rGO clearly present a high degree of oxidation with four functional groups corresponding to C–C (non-oxygenated ring C, 284.7 eV), C–O (C in C–O bonds, 286.1 eV), C=O (carbonyl C, 287.2 eV), and C–(O)–OH (carboxyl groups, 288.8 eV) [30]. For rGO moderately treated with UV ozone, the intensities of peaks assigned to C–O and C–(O)–OH increase slightly. The intensity of peaks assigned to C–O and C–(O)–OH increases more evidently than that of C=O. Therefore, rGO treated with UV ozone can validly induce hydrophilic group.

**Fig. 3:**
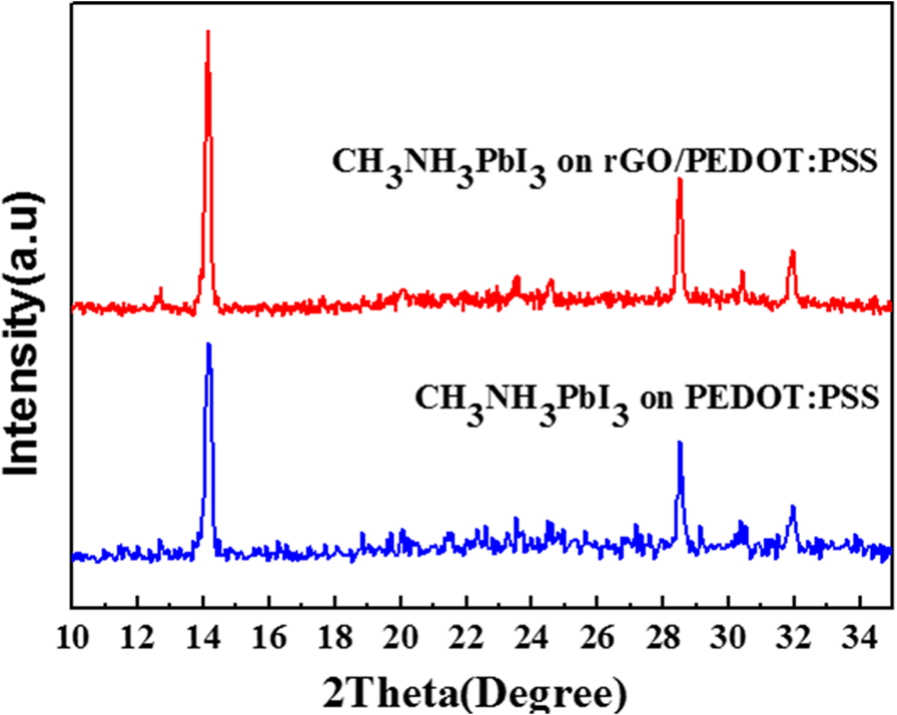
XRD patterns of the perovskite films on rGO/PEDOT:PSS and PEDOT:PSS

XRD spectra were obtained to investigate the crystallization structure of CH_3_NH_3_PbI_3_ layers. CH_3_NH_3_PbI_3_ thin films were spin coated on pristine PEDOT:PSS and rGO/PEDOT:PSS HTLs and subsequently annealed at 100 °C for 30 min. As shown in Fig. 3, both perovskite films exhibit similar features and show three peaks at 14.14°, 28.08°, and 31.86°, which are associated with the (110), (220), and (310) planes of perovskite crystals, respectively. Nevertheless, perovskite coated on hybrid rGO/PEDOT:PSS layer displays sharper diffraction peaks than those coated on original PEDOT:PSS, which suggested the improved crystallinity of perovskite on modified PEDOT:PSS layer.

**Fig. 4:**
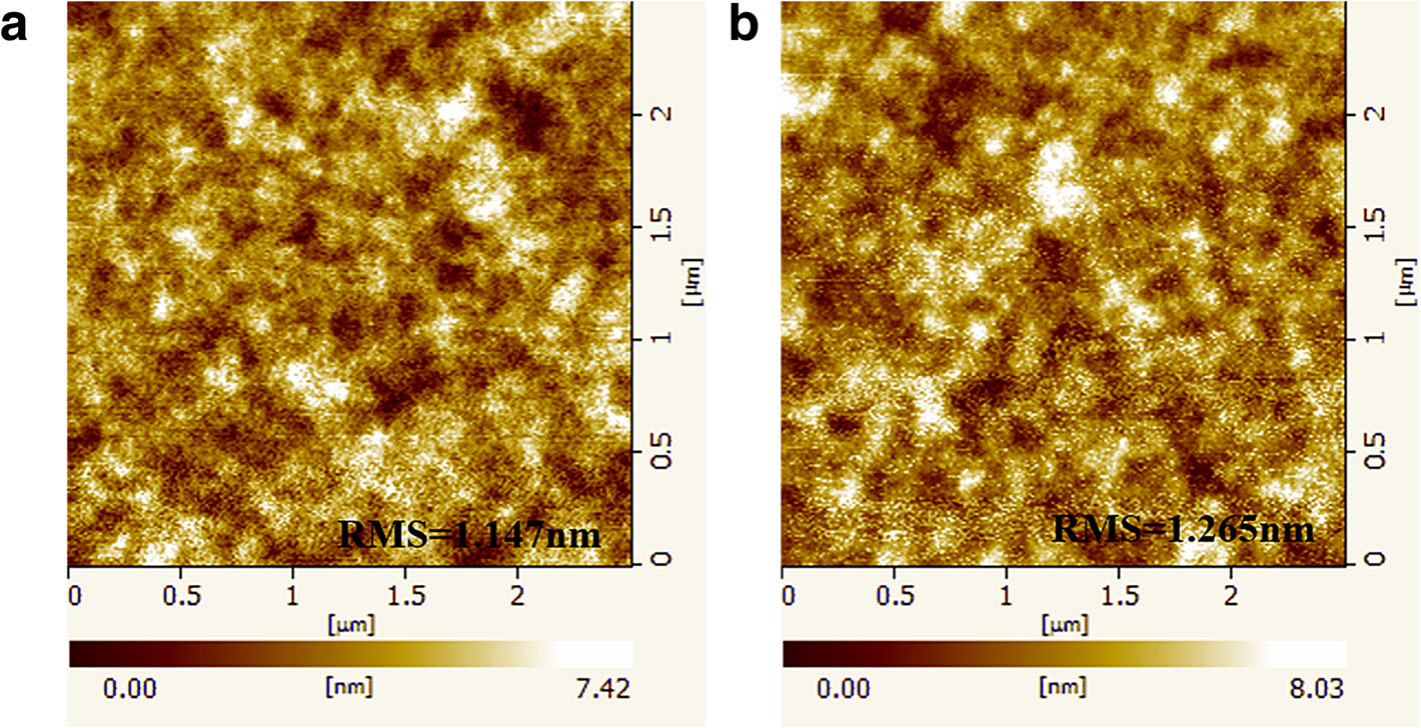
AFM top-view images of **a** pristine PEDOT:PSS and **b** rGO/PEDOT:PSS thin films: all images captured an area of 2.5 × 2.5 μm^2^

AFM was carried out to investigate the effect of rGO incorporation on PEDOT:PSS. Figure 4 shows the top-view AFM images of PEDOT:PSS and rGO/PEDOT:PSS thin films. These AFM top-view images of rGO/PEDOT:PSS thin films reveal no evident sign of rGO in the scanned area. This result is attributed to that the rGO is in the middle of PEDOT:PSS layer with a sandwich-like structure. In addition, the root-mean-square (RMS) roughness of pristine PEDOT:PSS layer is approximately 1.15 nm. The rGO/PEDOT:PSS thin films possess a RMS roughness of 1.27 nm. Previous literature reported [19] that slightly high substrate surface roughness is beneficial to perovskite crystallization process, and it causes large grain size and improved crystallinity, which is in agreement with the conclusion shown in Fig. 3.

**Fig. 5:**
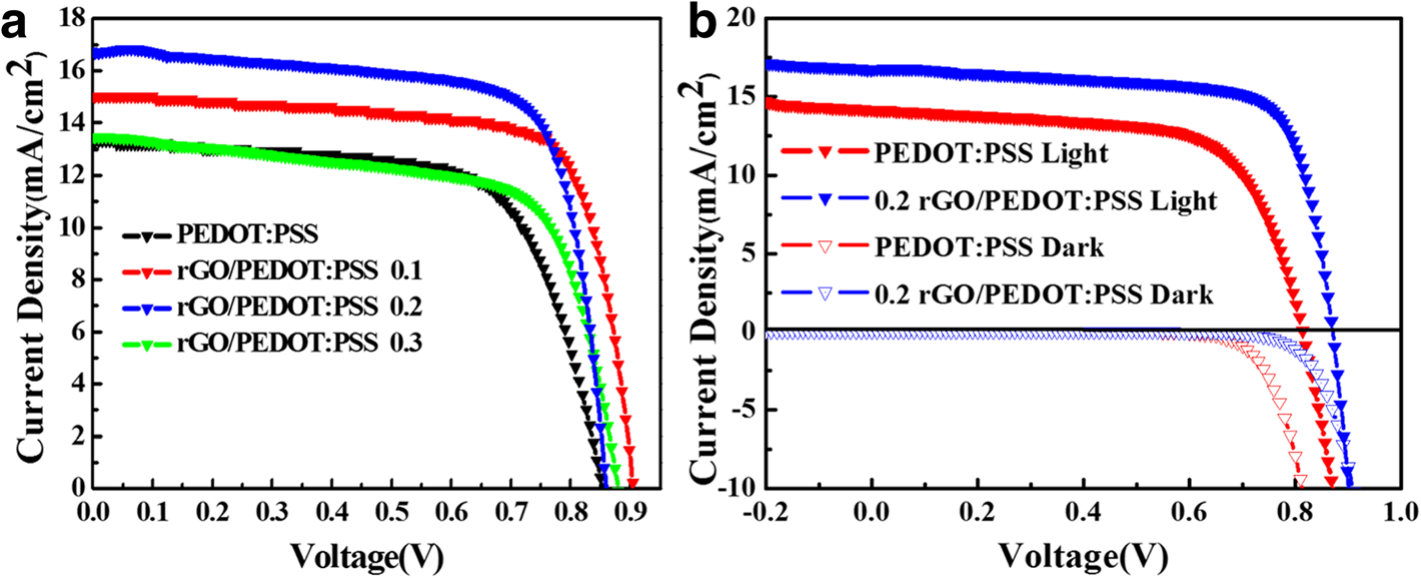
**a** J–V curves of the PSC with pristine PEDOT:PSS and PSCs with rGO/PEDOT:PSS at different volume ratios. **b** J–V curves of PSCs with pristine PEDOT:PSS as HTL (red lines) and PSCs (blue lines) with rGO/PEDOT:PSS (0.2 *v*/*v*) as HTL measured under simulated AM1.5 sunlight of 101 mW/cm^2^ irradiance (solid lines) and in the dark (dashed lines)

The rGO concentration in PEDOT:PSS is regulated so as to optimize the performance of PSCs. Figure 5a shows the J–V curves of the PSC with pristine PEDOT:PSS and PSCs with rGO/PEDOT:PSS at different volume ratios. PSCs with pristine PEDOT:PSS exhibit a *V*oc of 0.85 V, a *J*_SC_ of 13.29 mA/cm^2^, a FF of 66%, and a corresponding PCE of 8.48%. For PSCs with 0.1, 0.2, and 0.3 volume ratios of rGO/PEDOT:PSS as HTLs, the *V*_OC_ values are 0.90, 0.87, and 0.89 V, respectively. Correspondingly, the *J*sc is 15.04, 16.75, and 13.44 mA/cm^2^; the FF is 66, 75, and 73%, and 68%; and the PCE is 10.16, 10.75, and 8.16%, respectively. Overall, the most remarkable device with a *V*_OC_ of 0.87 V, a *J*_SC_ of 16.75 mA/cm^2^, a FF of 75%, and a PCE of 10.75% was observed in the PSCs incorporated with 0.2 *v*/*v* rGO/PEDOT:PSS as HTL. Both the *V*_OC_ and *J*_SC_ of the PSCs incorporated with 0.2 *v*/*v* rGO/PEDOT:PSS as HTL increase significantly compared with those of PSCs incorporated with pristine PEDOT:PSS as HTL. Consequently, approximately 27% enhancement was observed in the PSCs incorporated with 0.2 *v*/*v* rGO/PEDOT:PSS as HTL.

To understand the improved *V*_OC_ and *J*_SC_ for PSCs with rGO/PEDOT:PSS as HTL, Fig. 5b shows the J–V curves of PSCs with pristine PEDOT:PSS as HTL and PSCs with rGO/PEDOT:PSS (0.2 *v*/*v*) as HTL, respectively. The significantly increased value of *J*sc is mainly due to the decreased series resistor of the device. In addition, decreased dark current also contributes to the increment of the *J*sc of the devices according to a previous study [31–33]. To further elucidate the mechanism underlying the improvement of the device performance, the J–V curves of the devices in dark condition were also characterized. J–V measurement in the dark plays an important role in examining the diode properties of the solar cells [34]. Dark J–V measurements use electrical methods to inject carriers into the circuit rather than with photogenerated carriers to provide additional information about the cell for diagnostic purposes. The J–V curves of PSCs with pristine PEDOT:PSS as HTL and rGO/PEDOT:PSS as HTL measured in the dark are shown in Fig. 5b. The dark current value for PSCs with rGO/PEDOT:PSS as HTL is lower than that for PSCs with pristine PEDOT:PSS as HTL. This result indicated that the leakage current of the PSCs with rGO/PEDOT:PSS as HTL is suppressed. For solar cells, dark current includes reverse saturated current, thin film leakage current, and bulk leakage current. Therefore, many photogenerated charge carriers can flow through the device rather than being directly offset by dark current or shunting. Overall, the dark current is suppressed by the highly electrical conductive rGO-doped PEDOT:PSS HEL. Consequently, the *V*_OC_ and *J*_SC_ are improved, which fits with the data obtained from the dark J–V curves.

**Fig. 6:**
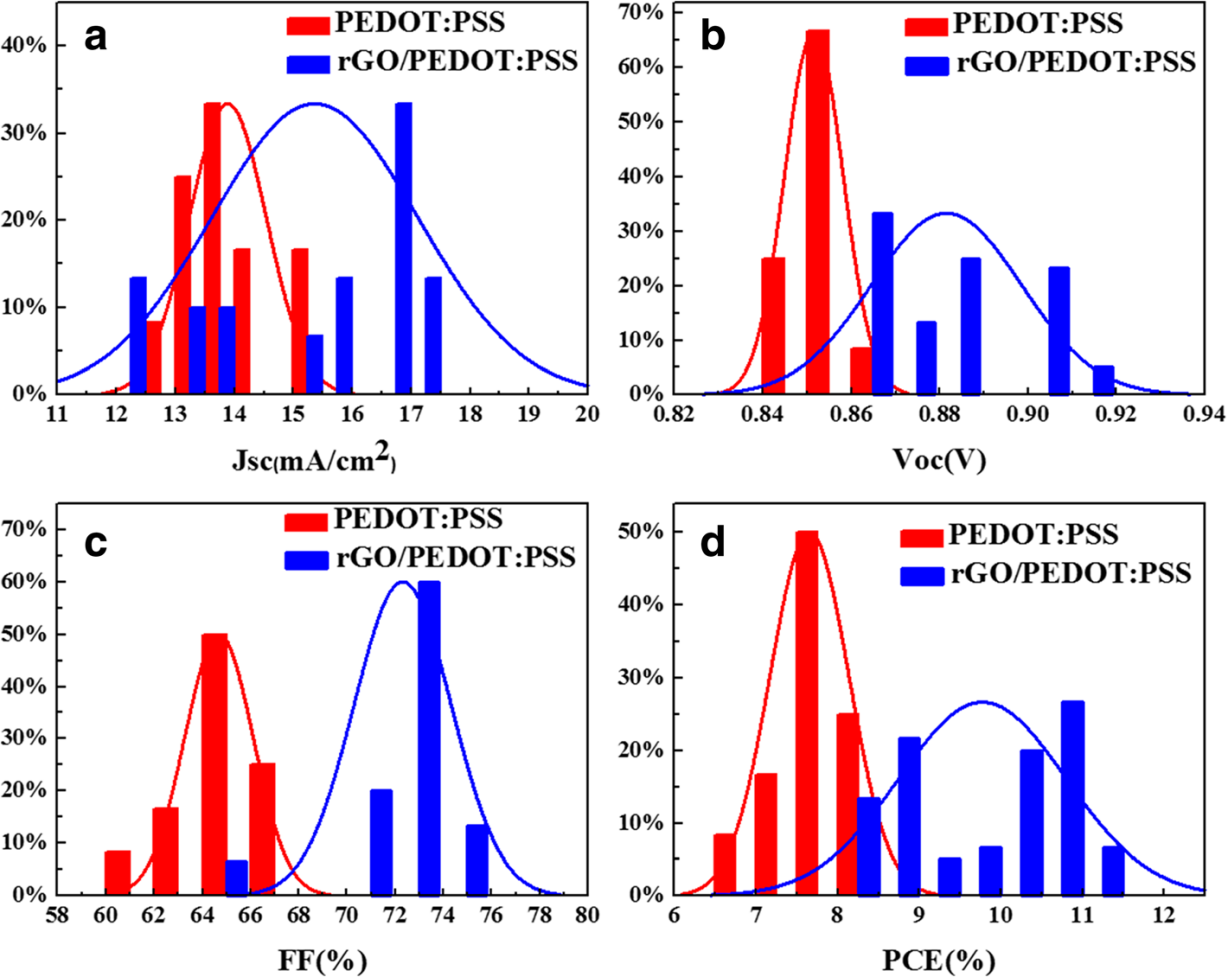
Histograms of statistical photovoltaic parameters **a** *V*_OC_, **b** *J*_SC_, **c** FF, and **d** PCE for PSCs with pristine PEDOT:PSS as HTL and rGO/PEDOT:PSS as HTL

Histograms of statistical photovoltaic parameters (*V*_OC_, *J*_SC_, FF, and PCE) for PSCs with pristine PEDOT:PSS as HTL and rGO/PEDOT:PSS as HTL are shown in Fig. 6. Statistical data were derived from a total of 60 devices. Most of the photovoltaic parameters are in agreement with Gauss distribution despite a few experimental data, as shown in the fitting curves in Fig. 6. According to the statistical data, the *V*_OC_, *J*_SC_, FF, and PCE of PSCs with pristine PEDOT:PSS as HTL are 0.85 ± 0.01 V, 13.88 ± 0.65 mA/cm^2^, 64.69 ± 1.41%, and 7.65 ± 0.48%, respectively. However, the *V*_OC_, *J*_SC_, FF, and PCE of PSCs with rGO/PEDOT:PSS as HTL are 0.88 ± 0.02 V, 15.25 ± 1.8 mA/cm^2^, 72.37 ± 2.03%, and 9.7 ± 1.04%, respectively. In brief, the *V*_OC_ presents no evident change. FF and *J*_SC_ increase significantly, which causes a 27% enhancement of efficiency. Intrinsically, the rGO increases the *J*sc and FF of the PSCs incorporated with rGO/PEDOT:PSS as HTL. Both *V*_OC_ and *J*_SC_ of the PSCs incorporated with 0.2 *v*/*v* rGO/PEDOT:PSS as HTL increase significantly compared with those of PSCs incorporated with pristine PEDOT:PSS as HTL. Consequently, approximately 27% enhancement is observed in the PSCs incorporated with 0.2 *v*/*v* rGO/PEDOT:PSS as HTL.

## Conclusions

We reported a simple and effective UV-ozone treatment method to obtain high-performance and solution-processable rGO. We also demonstrated the UV-ozone-treated rGO as an additive to modify the PEDOT:PSS as HTL for the fabrication of efficient PSCs. Solar cells based on treated rGO-doped PEDOT:PSS showed promising performance with a *V*_OC_ of 0.87 V, a *J*_SC_ of 16.75 mA/cm^2^, a FF of 75%, and a PCE of 10.75%. Furthermore, given the excellent surface morphology and enhanced hole mobility, a 27% photoelectric conversion efficiency enhancement was observed in the PSCs incorporated with 0.2 *v*/*v* rGO/PEDOT:PSS as HTL. The distinct advantages of solution-processable rGO provide a new possibility to achieve high-efficiency solar cells and other photoelectric devices.
